# THC induced similar physiological effects on HIV transgenic rats and their controls without affecting HIV-induced deficits in effortful motivation

**DOI:** 10.1186/s42238-025-00383-8

**Published:** 2026-01-02

**Authors:** Sunitha Vemuri, Samantha M. Ayoub, Arpi Minassian, Jared W. Young

**Affiliations:** 1 Department of Psychiatry, University of California San Diego, 9500 Gilman Drive MC 0804, La Jolla, San Diego, CA 92093-0804 USA; 2 Research Service, VA San Diego Healthcare System, San Diego, CA USA

**Keywords:** Phytocannabinoid, HAND, Apathy, Pain, Exploration

## Abstract

**Background:**

Cannabinoid*s* have proven to useful for attenuating adverse effects of HIV. People living with HIV use cannabis at higher rates, with evidence suggesting it alleviates physiological symptoms such as nausea, loss of appetite, etc.. Cannabis can alter physiology as well as essential behavioral functions such as motivation. This study investigated the effect of delta-9-tetrahydrocannabinol (THC), the main psychoactive component of cannabis, on physiological responses and motivation in HIV-1 transgenic (tg) rats and their controls.

**Methods:**

In Experiment 1, adult female and male HIV-1tg (*n* = 46) rats and their controls (wildtype littermates [WT] and Fischer344 [F344] rats; *n* = 87) were tested for acute THC-induced (0, 0.3, 3 mg/kg) physiological effects using the cannabinoid tetrad assay: 1) nociception, 2) body temperature, and 3) locomotor and exploratory behavior. In Experiment 2, adult female and male HIV-1tg (*n* = 58) rats and controls (*n* = 84) were tested in the Progressive Ratio Breakpoint Task (PRBT) to assess effortful motivation at baseline, after acute THC, then chronic (16 days) THC treatment (0, 0.3, 3 mg/kg). Data collected was analyzed using separate univariate ANOVA test with group (HIV-1tg, WT, F344), drug (0, 0.3, 3 mg/kg THC) and sex (female and male) as fixed factors. Bonferroni adjustments were used to correct for multiple comparisons.

**Results:**

THC (3 mg/kg) reduced nociception, temperature, and locomotor and exploratory activity across genotypes with some sex-dependent effects. Further, HIV-1tg and WT rats showed reduced motivation compared to F344 controls across PRBT testing timepoints. Acute 3 mg/kg THC reduced breakpoints but with no effects after chronic treatment.

**Conclusion:**

Hence, THC produces consistent physiological and motivational across HIV-1tg rats and their controls. Additionally, the HIV-1tg rat exhibited motivational deficits only when compared to the F344 but not WT controls, suggesting careful selections of control groups in future studies.

**Supplementary Information:**

The online version contains supplementary material available at 10.1186/s42238-025-00383-8.

## Background

People living with Human Immunodeficiency Virus (HIV; PWH) often experience a range of physiological and neurocognitive impairments (NCI), which persist even when viral load is suppressed by antiretroviral therapies and significantly impact daily functioning and quality of life (Tozzi et al. 2004; Nichols et al. 2019). As a result, many individuals seek alternative methods to manage their HIV symptoms. Cannabis use is prevalent among PWH, with rates of consumption reaching 70% (Costiniuk et al. 2019). Beyond recreational use, PWH report their cannabis use reduces common symptoms of HIV, including nausea, loss of appetite, and neuropathy (Dansak 1997; Fairfield et al. 1998), suggesting self-medication purposes.

Chronic exposure to viral HIV-proteins drives neuroinflammatory processes in the central nervous system (CNS) (Young et al. 2022; McArthur and Johnson 2020; Sinharay and Hammoud 2019) which are thought to contribute to HIV-related symptomology (Brody et al. 2025; Rackstraw 2011; Pedro et al. 2018; Chang et al. 2004). Cannabinoids, including Delta-9-tetrahydrocannabinol (THC) and Cannabidiol (CBD), have neuroprotective and anti-inflammatory properties via interactions with the endocannabinoid (eCB) system. Harnessing these mechanisms, cannabinoids have the potential to reduce neuroinflammation (Kaddour et al. 2022; Yndart Arias et al. 2023) and counter HIV-induced neuronal toxicity and associated neural damage (Esposito et al. 2002; DeMarino et al. 2022) to potentially alleviate HIV-induced physiological effects and NCI.

Experimental studies in PWH have already showed the utility of cannabinoid treatment in reducing HIV physiological complications such as HIV-associated anorexia and wasting syndrome (Bedi et al. 2010; Beal et al. 1995; Struwe et al. 1993; Haney et al. 2007; DeJesus et al. 2007). However, the impact of cannabinoids on HIV-associated NCI is less clear. Preclinical and clinical studies suggest cannabis, or its constituents, may mitigate HIV-induced NCI (Ayoub et al. 2024). Importantly, these beneficial effects appear dependent on the cognitive function tested and other modulating factors, including cannabis use patterns (e.g., frequency of use), and patient demographics (e.g., age) (Ayoub et al. 2024). For example, occasional, but not frequent cannabis use was associated with better global cognitive function in older PWH (Watson et al. 2023). On the other hand, chronic but not acute exposure to dronabinol, a synthetic compound akin to THC, slowed processing-speeds of PWH (Bedi et al. 2010). Other factors, such as age of cannabis use onset, are thought to influence cannabinoid effects on HIV-associated NCI but are difficult to control for in patient populations. This necessitates more experimental approaches, currently lacking in the literature. Hence, the impact of cannabinoids on HIV-associated NCI is not fully understood, and well-controlled animal studies can help guide more certain answers to HIV and cannabinoid interactions on behavior.

Unlike other rodent models of HIV, the HIV-1 transgenic (tg) rat carries an integrated HIV-1 genome (7 of 9 viral proteins), providing a reliable representation of PWH receiving effective antiretroviral therapy with suppressed viral replication, given this model is non-infectious (Reid et al. 2001). Importantly, HIV-1tg rats exhibit physiological and behavioral measures consistent with PWH, such as immune deficiency (Reid et al. 2001), sensorimotor gating deficits in both sexes (Jha et al. 2024; Roberts et al. 2021a), neuroinflammation and cognitive impairment (Li et al. 2021; Repunte-Canonigo et al. 2014; Ayoub et al. 2025). Hence, HIV-1tg rats are particularly valuable for investigating potential therapies for HIV-induced physiological effects and NCI. Since these rats are transgenic, the traditional control group would be their wildtype (WT) littermates. Given the chance of potential sporadic transgene insertion in WT rats however, age-matched Fischer 344 (F344) rats are also commonly used as controls, in addition to the use of WT littermates (Holmes 2003; Denton et al. 2019; Peng et al. 2010; Royal et al. 2007). The F344s provide the benefit of eliminating concerns about sporadic transgene insertion, though unlike WT littermates they cannot ensure consistent environmental exposures (e.g., shared housing and maternal care), which may also influence behavioral outcomes. Indeed, HIV-1tg rats show spatial memory deficits relative to both F344 and WT rats, with some differences, albeit minimal, found between control animals’ behavior (Vigorito et al. 2007; Lashomb et al. 2009). Hence, utilizing both F344 and WT littermate controls can help us isolate HIV-1 transgene effects in a manner that best controls for both background genetics and environmental factors that may influence behavior.

Importantly, before cannabinoid-induced cognitive outcomes can be interpreted in animal models of neuroHIV, it is critical to establish and control for potential physiological and basic behavioral effects they produce in the model, when compared to their controls. Indeed, despite their neuroprotective properties, cannabinoids like THC also induce physiological responses such as hypothermia, hypomotility, catalepsy, and antinociception – more commonly referred to as the cannabinoid tetrad (Little et al. 1988). The cannabinoid tetrad can inform eCB receptor function, as these behaviors are mediated through cannabinoid receptors, primarily cannabinoid-1 receptors (Metna-Laurent et al. 2017a). Similarly, THC has been shown to reduce motivation (Murray et al. 2022; Wardle et al. 2022), which could further complicate heightened apathy already seen in PWH (Castellon et al. 1998; Paul et al. 2005). Since basic physiological effects, such as motor impairment, and motivational factors such as reward-chasing, can influence cognitive performance (Edwards and Christie 2017; Stijntjes et al. 2017; Fervaha et al. 2014), it is essential to determine the influence of cannabinoids on such behaviors in HIV-1tg rats, in order to facilitate interpretations of cannabinoid-induced cognitive effects in the model. For example, HIV-1tg rats did not show THC-induced impairments on cognitive flexibility (Ayoub et al. 2025), nor sensorimotor gating (Roberts et al. 2021), as seen in their control animals – perhaps stemming from differences in eCB system function.

The current study sought to determine the effects of THC exposure on the basic physiological and motivational behaviors of HIV-1tg rats relative to their controls. To measure physiological responses, the cannabinoid tetrad behaviors of hypothermia, hypomotility, and antinociception were measured. To measure motivational behaviors, a separate group of animals were tested in the Progressive Ratio Breakpoint Task at baseline and again following acute and chronic THC exposure. Therefore, we tested the impact of THC on the behaviors above in the HIV-1tg rat model, and both WT and F344 controls, to help inform future studies which utilize this rat model of neuroHIV to explore cannabinoid-induced cognitive outcomes. Given the exploratory nature of this study, no formal hypotheses were proposed.

## Materials and methods

### Animals

As previously described, HIV-1tg rats were bred onto the Fischer 344 inbred rat strain, and both WT littermates and Fischer 344 rats served as controls (Ayoub et al. 2025). A total of 275 female and male rats were bred and raised in-house for these studies, with training and testing occurring in adulthood after 3 months of age. In Experiment 1, 133 rats were used (42% male), including 46 HIV-1tg rats, 47 WT littermates, and 40 Fischer 344 rats. In Experiment 2, 142 rats were tested (51% male), including 58 HIV-1tg rats, 48 WT littermates, and 36 Fischer 344 rats.

Animals were pair-housed in ventilated shoebox cages with standard environmental enrichment (plastic tube housing and nesting material; LWH: 15.5″ × 11.5″ × 8″) in a temperature-controlled room on a reversed light–dark cycle (7:00/19:00). Food and water were provided ad libitum, unless otherwise indicated. All behavioral testing began at least 2 h into the animal’s dark (active) phase and only occurred during their active period. Rats were maintained in a University of California San Diego (UCSD) animal facility that meets all federal and state requirements for animal care, and all procedures were approved by the Institutional Animal Care and Use Committee (IACUC) at UCSD.

### Drug preparation

THC dissolved in ethanol was obtained from the National Institute on Drug Abuse. The ethanol was evaporated under a stream of dry nitrogen, and the residue was dissolved to final concentrations of 0.3 and 3 mg/mL in a vehicle (VEH) consisting of 7.5% Tween-80 and 7.5% propylene glycol (Sigma-Aldrich, St. Louis, MO, USA) in saline. Rats were injected at 1 mL/kg resulting in doses of 0.3 and 3 mg/kg. Doses were chosen based on previous studies demonstrating their efficacy in producing measurable cannabinoid effects in rats (Klein et al. 2011; Malone et al. 2009; Panlilio et al. 2012; Nguyen et al. 2016), with the high-dose also representing typical daily THC consumption in daily users (Borodovsky et al. 2025; Kerr and Ye 2022; Larsen et al. 2023).

### Experiment 1: THC impact on the cannabinoid tetrad assay

#### Hot water tail flick assay

Nociception was assessed using a hot water tail flick test, consistent with prior cannabinoid studies (Moore and Weerts 2022). The apparatus consisted of a glass beaker holding water heated up to and maintained at 54 °C. Baseline measures of nociception and body temperature were first collected for each rat. To assess nociception, rats were immobilized in a handhold, and the distal 3/4ths of their tails were immersed in a warm water bath. The time taken (in seconds) for each rat to remove its tail from the water was recorded. Tail flick responses were measured twice per rat and averaged, both at baseline (pre-drug) and following THC administration (post-drug). If a rat did not perform the tail flick within 10 s, it was removed from the water bath to prevent physical injury.

#### Rectal temperature recordings

Body temperatures were recorded using a Vicks Baby Rectal Thermometer. Rats were immobilized, and a baby thermometer was inserted rectally. Temperature readings (in Fahrenheit) were measured twice and averaged to determine pre- and post- drug scores.

#### Behavioral Pattern Monitor (BPM)

Locomotor and exploratory activity was recorded using the rodent BPM, consistent with prior studies (Jha et al. 2024). The BPM apparatus is a computer-monitored chamber (65 × 96 cm) equipped with 8 wall and 3 floor holes that serve as discrete stimuli for rodents to investigate. Infrared beams allow for the tracking of nose-poking behavior and locomotor responding within 9 defined chambers. This technique enables the collection of exploratory and locomotor responses such as counts, transitions, pokes, rears, spatial d, and distance covered, all previously described elsewhere (Ayoub et al. 2023). Spatial d provides a measure of locomotor patterns on a scale of 1 to 2, with values nearing 1 representing linear movement and values closer to 2 representing sporadic movement (Young et al. 2016). Closed monitors enable studying the effects of psychoactive drugs of locomotor and investigatory behavior without external factors such as light or noise interacting.

#### Experimental timeline

**Fig. 1:**
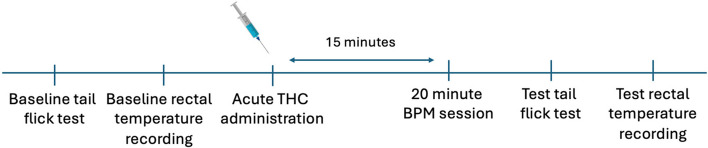
Testing timeline of the tetrad analysis. Experimental timeline. Baseline tail flick time and rectal temperatures were recorded then rats were injected with THC. Following 15 min from drug administration, subjects were then placed in the BPM for 20 min. Tail flick timings and rectal temperatures were recorded once more following BPM assessment to determine drug-induced changes

The timeline of assessment is illustrated in Fig. 1. Immediately after baseline assessment, rats were injected with THC intraperitoneally (i.p.) at doses of 0.3 mg/kg, 3 mg/kg, or vehicle. Fifteen minutes after drug administration, rats were placed in the BPM for 20 min to measure locomotor and exploratory behavior. After the BPM session, tail flick reaction times and rectal temperatures were reassessed within the next five minutes.

### Experiment 2: THC impact on effortful motivation as measured by the Progressive Ratio Breakpoint Task

The experiment utilized 5-choice operant chambers (50 cm × 50 cm × 50 cm, Med Associates Inc., St. Albans, VT, USA) which were housed in larger sound-attenuating cabinets designed to reduce external noise and provide ventilation through built-in fans. Within the operant chambers, nosepoke holes fitted with LED lights were used to signal stimulus presentation and infrared beams monitored the rats’ responses. Correct responses triggered the delivery of 40 μl of strawberry milkshake reward (Nesquik in nonfat milk) into an illuminated reward magazine located on the opposite wall. The reward magazine featured an LED light to indicate reward delivery and infrared beams to detect when the reward was collected. All responses and stimulus outputs were controlled via the SmartCtrl Package (8-In/16-Out), with additional interfacing provided by MED-PC for Windows (Med Associates Inc., St. Albans, VT). Custom programming was used to log data such as nosepoke responses, breakpoints, and other behavioral metrics as described previously (Amitai et al. 2019).

**Fig. 2:**
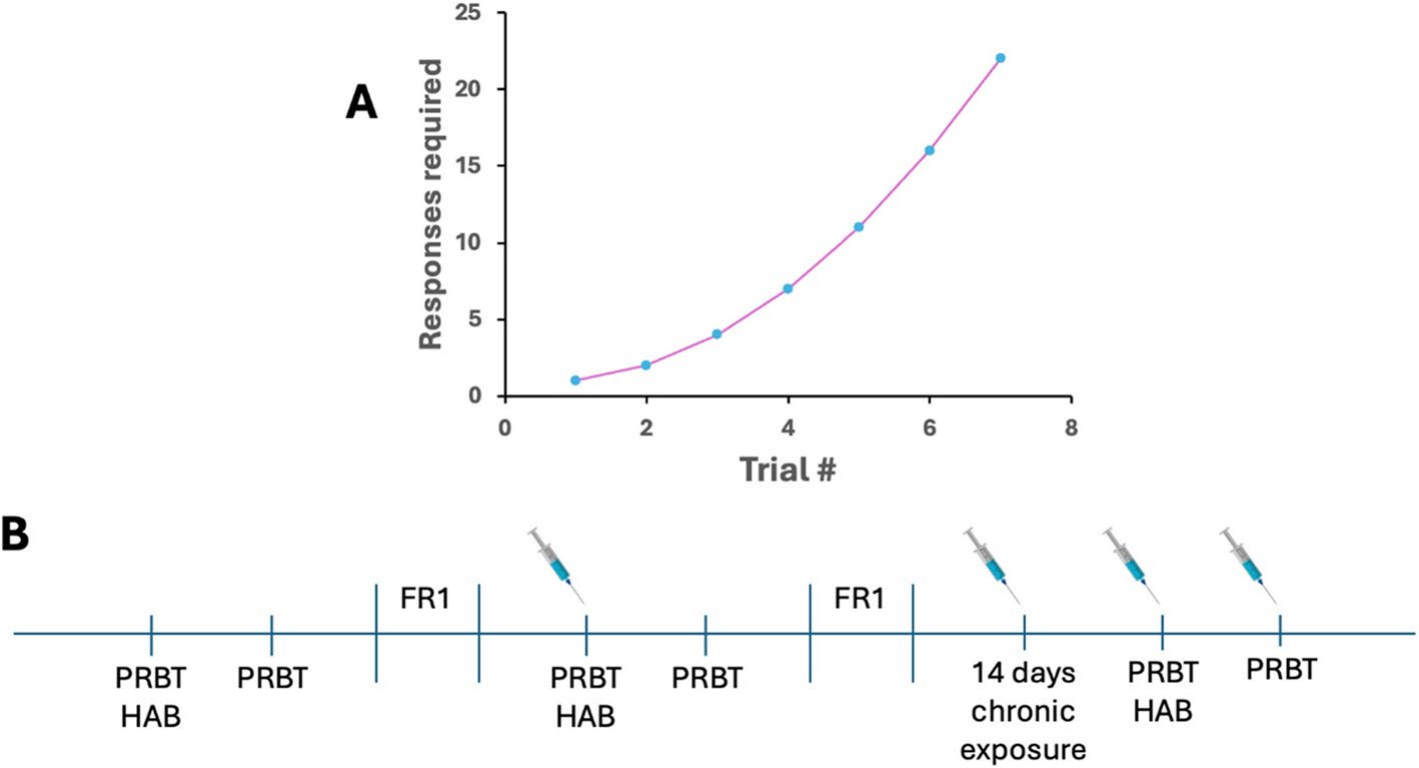
Schedule and testing timeline for assessing effortful motivation in the Progressive Ratio Breakpoint Task (PRBT). **A** Number of responses required for each subsequent reward in the PRBT task. **B** Animals were tested in the PRBT at baseline, following acute THC administration, and following 16 days of chronic THC. Each PRBT testing session was preceded by a test habituation session (HAB) in which animals were run on an FR1 schedule using only the nosepoke holes designated for testing

Subjects were maintained at ~ 85% baseline body weight while water was provided ad libitum (except when in testing chambers). Rats were trained in operant chambers to assess their motivation to obtain strawberry milkshake reward at three timepoints: baseline, post-acute THC exposure, and post-chronic THC exposure. Before training, rats were habituated to reward delivery in testing chambers with 40 μl milkshake delivery in the magazine at 15 s intervals for 30-min session. Once rats reached a minimum of 50 trials, they were moved to a Fixed Ratio (FR1) 1 schedule where a nosepoke was rewarded with the reward delivery. FR1 sessions ran 30 min until subjects performed 70 + trials per session for two consecutive sessions after which they were maintained on this schedule twice a week until testing to prevent overtraining. After stable choice reward was attained in all subjects, the task was switched to the Progressive Ratio Breakpoint Task (PRBT). During this 60-min session, rats were required to participate in a series of nosepoke trials where the number of responses required for each subsequent reward increased progressively (1, 2, 4, 7. 11. 16. 22, etc.; Fig. 2A). Breakpoints were recorded as the highest trial the rat completed before ceasing to respond.

#### Experimental timeline

Following baseline training, rats were injected i.p. with THC (0.3 mg/kg, 3 mg/kg) or VEH at acute and chronic time points. Rats were placed into operant chambers 30 min post THC injection to measure the breakpoint for each subject performing the PRBT test. Following a week after acute THC exposure, rats were injected with their assigned THC dose for a total of 16 days. During these chronic injections, rats were maintained on a FR1 operant schedule twice a week to maintain response behavior. On the 16th day of chronic THC exposure, rats were assessed 30 min after their last injection to measure the breakpoint following long-term exposure to THC (Fig. 2B).

### Statistical analyses

Tetrad and PRBT data were analyzed using separate univariate ANOVA test with group (HIV-1tg, WT, F344), drug (0, 0.3, 3 mg/kg THC) and sex (female and male) as fixed factors. Bonferroni adjustments were used to correct for multiple comparisons. Statistical analyses were performed using IBM SPSS Statistics v 29 (Armonk, NY, USA). Graphs were constructed using GraphPad Prism 9 (San Diego, CA, USA). The significance level for all analyses were set to *p* < 0.05, though trends are reported where observed (*p* < 0.1).

## Results

### Expt. 1: Physiological responses to acute THC were consistent across HIV-1tg rats and their controls in the cannabinoid tetrad assay

#### Nociception

**Fig. 3:**
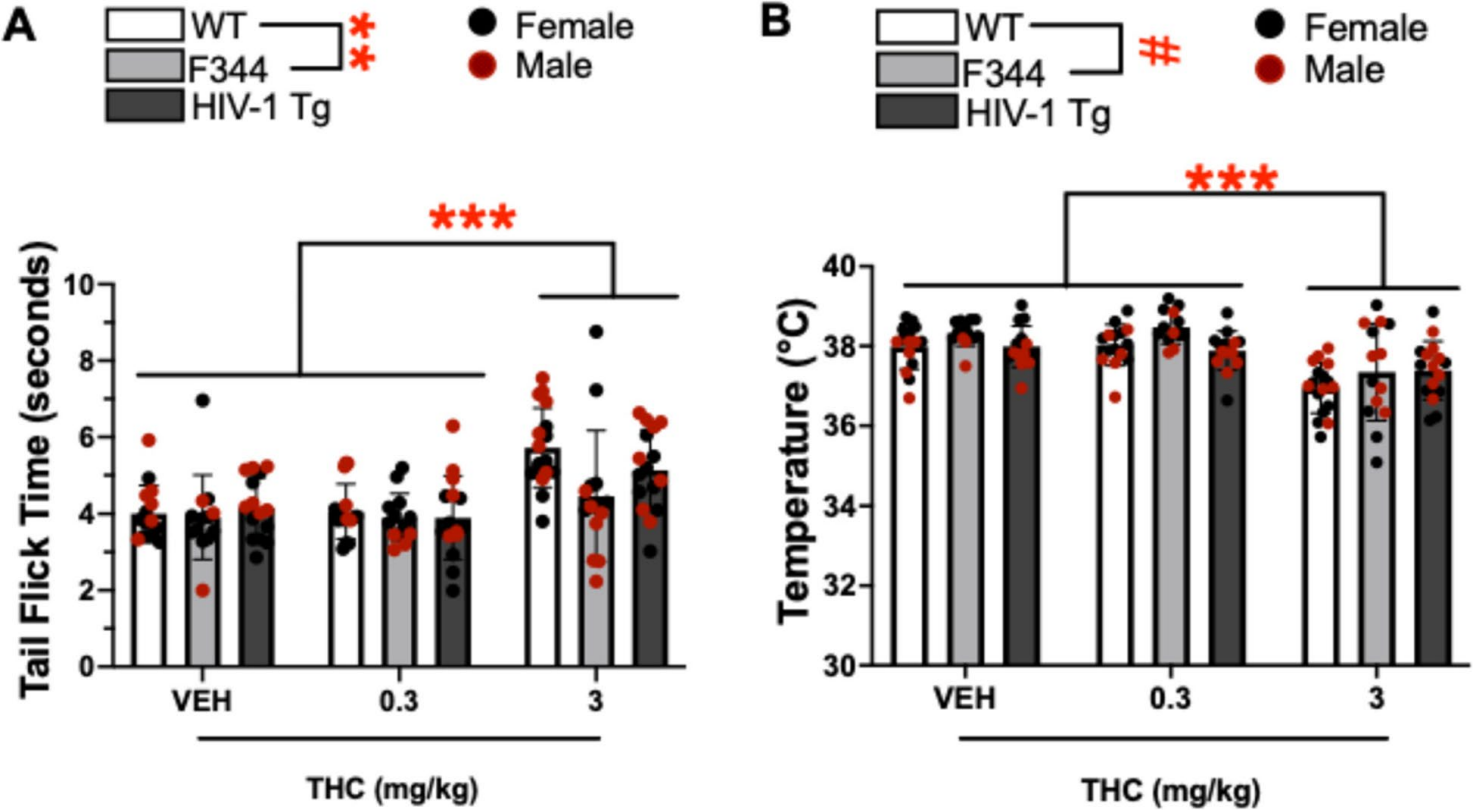
THC significantly increased tail flick time and decreased body temperature in both controls and tg animals. **A** Tail flick times and (**B**) Rectal body temperature recorded in WT, F344 (controls), and HIV-1 transgenic (tg) rats at acute exposure to vehicle (VEH), 0.3 mg/kg, and 3 mg/kg THC. THC (3 mg/kg) slowed the tail flick time (**A**) and dropped body temperature (**B**) irrespective of group relative to 0.3 mg/kg- and vehicle -treated rats. Bar plots represent group means, and individual points are shown ± S.E.M. Black data points represent female subjects while red points represent males. Significant main effects are indicated by **p < 0.01 or ***p < 0.001 and trending effects are indicated by #*p* < 0.1

A main effect of THC was observed on tail flick time outcome in the tail flick assay (F(2,115) = 22.879, *p* < 0.001; Fig. 3A). Post hoc analyses revealed that 3 mg/kg THC increased tail flick time relative to vehicle and 0.3 mg/kg THC (*ps* < 0.001). No main effect of sex, nor interaction between THC and sex was observed (F < 1.692, ns). A main effect of group was also observed on tail flick time (F(2,115) = 5.414, *p* = 0.006), with post hoc analyses revealing that WT rats had a higher tail flick time than F344 rats (*p* = 0.004). This effect appeared dependent on sex (group x sex: [F(2,115) = 15.051, *p* < 0.001]; Fig S1), where F344 rats had lower tail flick times relative to the other groups in males only (*ps* < 0.001) while F344 rats tended to have higher tail flick times relative to TG rats (*p* = 0.097). Further group comparisons revealed males had higher tail flick time relative to females in WT (*p* < 0.001) and TG (*p* = 0.002) groups, whereas females had higher tail flick times relative to males within the F344 group (*p* < 0.001).

#### Body temperature

A main effect of THC was observed on body temperature in rectal temperature recordings (F(2,115) = 24.672, *p* < 0.001; Fig. 3B). Post hoc analyses revealed that 3 mg/kg of THC reduced body temperature relative to 0.3 mg/kg THC and vehicle (*ps* < 0.001). THC did not interact with group (F < 1.026, ns). A main effect of group was also observed (F(2,115) = 3.065, *p* = 0.050), with post hoc analyses revealing that F344 rats had lower body temperature relative to WT rats (*p* = 0.046). A THC x sex interaction suggested the effect of THC was more pronounced in females (THC x sex: F(2,115) = 6.502, *p* = 0.002; Fig S2). Post hoc analyses revealed that 3 mg/kg THC reduced body temperature relative to 0.3 mg/kg THC and vehicle in females only (*ps* < 0.001) while 3 mg/kg THC tended to reduce body temperature relative to 0.3 mg/kg THC in males (*p* = 0.069). Further group comparisons revealed that males had lower body temperature relative to females when treated within VEH (*p* = 0.012) or 0.3 mg/kg THC (trend; *p* = 0.074), though females had lower temperatures than males when treated with 3 mg/kg THC. No main effect of sex or interactions of sex with group, or THC and group were observed (F < 2.176, ns).

#### Locomotor and exploratory responding

**Fig. 4:**
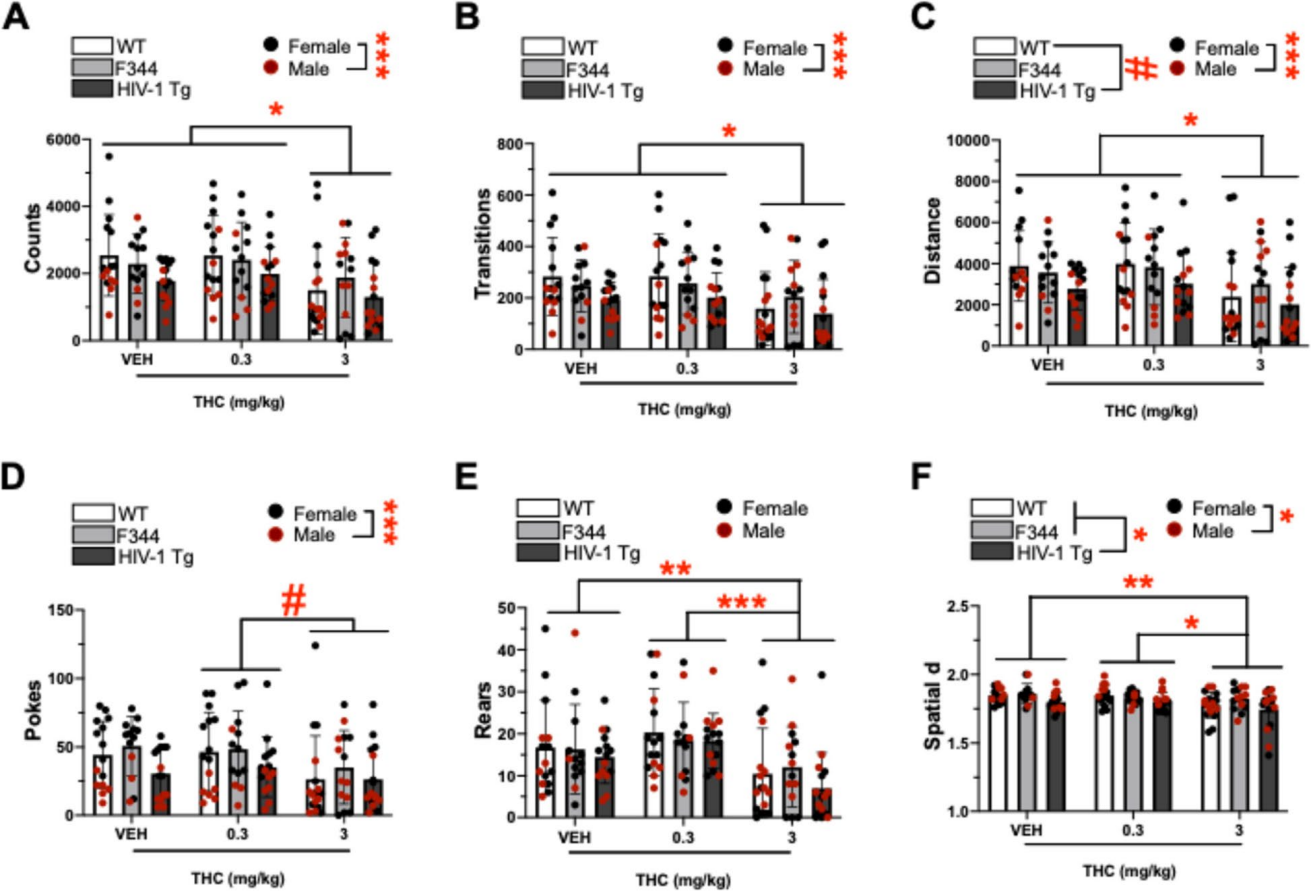
THC significantly reduced counts, transitions, distance, pokes, rears, and spatial d in both controls and tg animals. **A** Counts, **B** Transitions, **C** Distance travelled, **D** Pokes, **E** Rears and, **F** Spatial d recorded in WT, F344 (controls), and HIV-1 transgenic (tg) rats at acute exposure to vehicle (VEH), 0.3 mg/kg, and 3 mg/kg THC. THC (3 mg/kg) reduced counts (**A**), lowered transitions (**B**), lowered distance travelled (**C**), lowered pokes (**D**), reduced rearing behavior (**E**), and decreased spatial d (**F**) irrespective of group relative to 0.3 mg/kg- and/or vehicle (0)-treated rats. Bar plots represent group means, and individual points are shown ± S.E.M. Black data points represent female subjects while red points represent males. Significant main effects are indicated by *p < 0.05, **p < 0.01, or ***p < 0.001 and trending effects are indicated by #*p* < 0.1

A main effect of THC on counts was observed in the BPM (F(2,115) = 5.452, *p* = 0.005; Fig. 4A). Post hoc analyses revealed that 3 mg/kg THC reduced counts relative to 0.3 mg/kg THC (*p* = 0.011) and vehicle (*p* = 0.029). THC did not interact with sex, or group, or sex and group (F < 1.632, ns). A main effect of sex was observed (F(1,115) = 17.827, *p* < 0.001; Fig S3A), with post hoc analyses revealing that females had higher counts compared to males. Additionally, a trending effect of group was observed (F(2,115) = 2.617, *p* = 0.077), but post hoc analyses revealed no significant group differences. There were no group and sex interactions (F < 2.327, ns).

A main effect of THC on transitions was observed in the BPM (F(2,115) = 4.460, *p* = 0.014; Fig. 4B). Post hoc analyses revealed that 3 mg/kg THC decreased transitions relative to 0.3 mg/kg THC (*p* = 0.032) and vehicle (*p* = 0.042). THC did not interact with sex, or group, or sex and group (F < 1.294, ns). A main effect of sex was also observed (F(1,115) = 15.338, *p* < 0.001; Fig S3B), with post hoc analyses revealing that females had higher transitions than males. Moreover, a trending effect of group was observed on transitions (F(2,115) = 2.799, *p* = 0.065), but post hoc analyses revealed no significant group differences. This trending group effect appeared dependent on sex (group x sex: (F(2,115) = 2.575, *p* = 0.081), with post hoc analyses revealing that WT rats had higher transitions relative to TG rats in females only (*p* = 0.045). Further group comparisons revealed that females had higher transitions than males in WT (*p* < 0.001) and TG (*p* = 0.015) groups only.

A main effect of THC was observed on distance travelled in the BPM (F(2,115) = 4.550, *p* = 0.013; Fig. 4C). Post hoc analyses revealed that 3 mg/kg THC decreased distance travelled relative to 0.3 mg/kg THC (*p* = 0.036) and vehicle (*p* = 0.033). THC did not interact with sex, or group, or sex and group (F < 1.267, ns). A main effect of sex was also observed (F(1,115) = 14.606, *p* < 0.001; Fig. S3C), with post hoc analyses revaling that females travelled a higher distance relative to males. Additionally, a trending effect of group was also observed (F(2,115) = 3.000, *p* = 0.054), with post hoc analyses revealing that WT rats tended to travel higher distances than TG rats (*p* = 0.080). A trending group x sex interaction was also observed (F(2,115) = 2.438, *p* = 0.092), with post hoc analyses revealing that WT rats travelled higher distances relative to TG rats in females only (*p* = 0.040). Further group comparisons revealed that females travelled higher distances than males in WT (*p* < 0.001) and TG (*p* = 0.017) groups only.

A trending effect of THC on pokes was observed in the BPM (F(2,115) = 3.052, *p* = 0.051; Fig. 4D). Post hoc analyses revelaed that 3 mg/kg THC tended to decrease pokes relative to 0.3 mg/kg THC (*p* = 0.067). A main effect of sex was observed (F(1,115) = 54.159, *p* < 0.001; Fig S3D), with post hoc analyses revealing that females had higher pokes compared to males. The effect of THC appeared dependent on sex (drug x sex: [F(2,115) = 3.396, *p* = 0.037]), with post hoc analyses revealing that 3 mg/kg THC reduced pokes relative to 0.3 mg/kg THC (*p* = 0.002) and vehicle (*p* = 0.008) in females only. Further group comparisons revealed that males had decreased pokes relative to females across VEH and THC doses (*ps* < 0.05–0.001). No main or interactive effects of group were observed (F < 2.254, ns).

A main effect of THC was observed on rearing behavior in the BPM (F(2,115) = 10.527, *p* < 0.001; Fig. 4E). Post hoc analyses revealed that 3 mg/kg THC reduced rearing behavior relative to 0.3 mg/kg THC (*p* < 0.001) and vehicle (*p* = 0.009). No other main or interactive effects were observed on rearing behavior (F < 1.695, ns).

A main effect of THC was observed on Spatial d in the BPM (F(2,115) = 5.889, *p* = 0.004; Fig. 4F). Post hoc analyses revealed that 3 mg/kg THC decreased spatial d (more linear movement patterns) relative to 0.3 mg/kg THC (*p* = 0.025) and vehicle (*p* = 0.007). THC did not interact with sex, or group, or sex and group (F < 0.821, ns). A main effect of group was also observed (F(2,115) = 5.389, *p* = 0.006), with post hoc analyses revealing that TG rats had lower spatial d (more linear movement patterms) when compared to WT (*p* = 0.022) and F344 (*p* = 0.015) rats. Additionally, a main effect of sex was also observed (F(1,115) = 6.520, *p* = 0.012; Fig S3F), with post hoc analyses revealing that males had lower spatial d relative to females. No interaction between group and sex was seen (F < 2.176, ns).

### Expt. 2: HIV-1tg rats exhibit reduced motivation in the PRBT, with acute and chronic THC effects consistent across HIV group

#### Baseline assessment of HIV-1tg rats in the PRBT

**Fig. 5:**
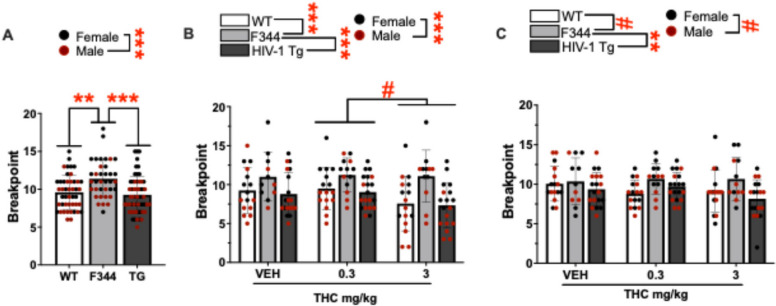
Acute THC reduced breakpoint in the Progressive Ratio Breakpoint Test (PRBT) while chronic exposure to THC did not have any effects irrespective of group or sex. Breakpoints in WT, F344 (controls), and HIV-1 transgenic (tg) rats at (**A**) Baseline, **B** post acute exposure to THC and, **C** post chronic exposure to THC relative to vehicle (VEH). **A** F344 rats had higher breakpoints relative to WT and tg animals irrespective of sex. **B** THC (3 mg/kg) reduced breakpoints relative to low dose (0.3 mg/kg). **C** Chronic THC exposure had no effects irrespective of group or sex. Bar plots represent group means, and individual points are shown ± S.E.M. Black data points represent female subjects while red points represent males. Significant main effects are indicated by **p < 0.01, or ***p < 0.001 and trending effects are indicated by #*p* < 0.1

A main effect of group was observed on baseline breakpoint (F(2,120) = 8.898, *p* < 0.001; Fig. 5A). Post hoc analyses revealed that F344 rats had higher breakpoints than WT (*p* = 0.007) and TG (*p* < 0.001) rats. A main effect of sex was observed (F(1,120) = 22.776, *p* < 0.001) with post hoc analyses revealing that females had higher breakpoints relative to males. No interactions between group and sex were seen (F < 0.222, ns).

#### Acute effects of THC in HIV-1tg rats in the PRBT

A main effect of group was observed on breakpoint during acute testing (F(2,123) = 12.105, *p* < 0.001; Fig. 5B). Post hoc analyses revealed that F344 rats had higher breakpoints relative to WT (*p* = 0.001) and TG (*p* < 0.001) rats. Group did not interact with THC, or sex, or THC and sex (F < 0.817, ns). A trending effect of THC was observed (F(2,123) = 2.958, *p* = 0.056) with post hoc analyses revealing that 3 mg/kg THC tended to reduce breakpoints relative to 0.3 mg/kg THC (*p* = 0.059) but not relative to vehicle (*p* > 0.1). A main effect of sex was also observed (F(1,123) = 41.149, *p* < 0.001), with post hoc analyses revealing that females had higher breakpoints than males. No interaction between sex and THC was observed (F < 1.201, ns).

#### Chronic effects of THC in HIV-1tg rats in the PRBT

A main effect of group was observed on breakpoint during chronic testing (F(2,122) = 4.656, *p* = 0.011; Fig. 5C). Post hoc analyses revealed that F344 rats had higher breakpoints than TG rats (*p* = 0.010) and tended to have higher breakpoints than WT rats (*p* = 0.069). No main or interactive effects of THC were observed (F < 1.347, ns). A trending effect of sex was observed (F(1,122) = 3.468, *p* = 0.065), with post hoc analyses revealing that females tended to have higher breakpoints than males. No interaction between group and sex was observed (F < 1.430, ns).

## Discussion

We observed that acute THC (3 mg/kg) produced cannabinoid tetrad behaviors of reduced nociception, hypothermia, and hypolocomotion across HIV-1tg rats and their controls, indicating that the HIV-1 transgene does not affect THC’s basic physiological effects. Further, HIV-1tg rats consistently exhibited significant motivational deficits in the PRBT relative to standard controls (F344 rats), with THC producing no effect on motivation irrespective of genotype. Importantly, the inclusion of both WT littermates and F344 rats captured notable differences between these commonly utilized control groups of the HIV-1tg rat model.

Our findings revealed group differences in the BPM between HIV-1tg rats and the control groups, as part of the cannabinoid tetrad. HIV-1tg rats tended to exhibit reduced distance traveled compared to WT controls, but not F344 controls, indicating locomotor deficits relative only to one control group. Consistently, prior studies reported marginal hypoactivity in the HIV-1tg rat relative to F344 controls in the BPM (Roberts et al. 2021b; Jha et al. 2024). Taken together, the HIV-1tg rat model may exhibit modest hypolocomotor behavior when compaired to either control animal, not always captured in separate cohorts. Impaired spatial d in HIV-1tg rats was also observed relative to both controls, an effect not previously reported (Roberts et al. 2021b). Reduced spatial d is observed in mania patients (Minassian et al. 2010; Paulus et al. 2007; Perry et al. 2009) and animal models of bipolar mania (Perry et al. 2009), albeit alongside enhanced locomotor and exploratory responding. In contrast, PWH may exhibit reduced locomotor activity and more linear movement patterns (data unpublished). This reduction in locomotor and exploratory behavior is consistent with prior evidence of dopaminergic dysfunction and altered exploratory behavior in the HIV-1tg model (June et al. 2009; Moran et al. 2013).

HIV-1tg rats also exhibited reduced breakpoints for sweet reward in the PRBT, which is consistent with prior observations of diminished reinforcing efficacy of, and sensitivity to, sucrose in HIV-1tg rats vs. F344 controls (McLaurin et al. 2021a). Consistently, reduced breakpoints for cocaine self-administration in HIV-1tg rats was seen relative to F344 controls (McLaurin et al. 2021a). Lower motivation is consistent with reported apathy in PWH (Castellon et al. 1998; Kamat et al. 2012; Rabkin et al. 2000) and may be driven by altered prefrontal cortex and basal ganglia function (Levy and Dubois 2005), areas which are affected by HIV (McIntosh et al. 2018; McLaurin et al. 2021b; Berger et al. 2000; Chang et al. 1999). Prior studies report that detection of apathy in PWH can aid in identifying functional impairment, as higher apathy in PWH were associated with greater declines in instrumental activities of daily living as well as higher cognitive complaints (Kamat et al. 2012). Another study reported that apathy was associated with lower health-related quality of life in PWH independent of variability in mental and physical quality of life (Kamat et al. 2016). Determining the mechanism(s) underlying such apathy are vital. HIV-1tg rat exhibit dopaminergic dysregulation, with elevated dopamine uptake in the prefrontal cortex (PFC) and striatum, plus region-specific alterations of DAT expression in the PFC and striatum (Zhu et al. 2016). Older HIV-1tg rats show dopaminergic neuronal loss and dysfunction, likely due in part to elevated nitrosative stress (Shah et al. 2019). Given that dopamine is essential for motivation—particularly through its role in encoding incentive salience and encouraging goal-directed behavior (Bromberg-Martin et al. 2010; Wise 2004)—such dysregulation is likely to impair motivational processes. THC tends to increase brain dopamine, though long term exposure may instead suppress dopamine neurotransmission (Bloomfield et al. 2016). Thus, theoretically acute THC could have prevented HIV-1tg rat motivational deficits, and chronic THC could have further worsened motivation in the HIV-1tg rats, not observed. Other neurotransmitter systems (e.g., cholinergic) may instead drive HIV-1tg rat motivational deficits, or perhaps route of administration differences between our study (i.e., lower doses, i.p. injection) and studies that report in vivo THC-induced increases of dopamine in rats led to these lack of findings. Our data imply that the HIV-1tg rat may be an appropriate model to further explore the neurobiological drivers of reduced motivation and apathy as seen in PWH (Castellon et al. 1998; Paul et al. 2005; Bryant et al. 2015; Kamat et al. 2016a, 2016b).

Neither acute nor chronic administration of THC affected breakpoints at the doses tested. Although THC-did not alter the motivational deficits of HIV-1tg rats in our study, the absence of drug x group interactions indicates that—similar to tetrad effects (see below) – the HIV-1 transgene did not differentially alter the motivational impact of THC in the PRBT. Hence, THC may not exacerbate HIV-induced apathy, as seen in PWH (Castellon et al. 1998; Kamat et al. 2012; Rabkin et al. 2000). Importantly, our results suggest notable differences between control groups, with F344 controls displaying higher motivation compared to WT littermate controls. This difference indicates potential strain-related variations in reward-seeking behavior, although less pronounced at chronic testing, which could influence the interpretation of HIV-1tg deficits. While F344 rats are often used due to their genetic consistency (Holmes 2003; Denton et al. 2019; Peng et al. 2010; Royal et al. 2007), WT littermates more accurately represent the environmental background of HIV-1tg rats, and are also common controls (Vigorito et al. 2007; Royal et al. 2012; Lassiter et al. 2009; Joshi and Guidot 2011). In addition to group differences between F344 and WT controls in nociception and rectal temperatures (Fig. 3), and locomotor activity (Fig. 4C), these data highlight clear behavioral differences between commonly used control animals for the HIV-1tg rat model. Such differences may have stemmed from sporadic transgene insertion into the genome of HIV-1 WT littermates, or from differential homecage environments of the HIV-1tg and WT rats relative to F344 rats. Hence, careful consideration of control group selection should be taken when utilizing the HIV-1tg rat model and future research should further investigate these control strain differences to better delineate the contributions of genetic background to behavioral outcomes.

Despite these basic differences between groups, THC produced the cannabinoid tetrad behaviors of antinociception, hypothermia, and hypolocomotion across all animals, a phenotype elicited upon acute administration of cannabinoid-1 receptor (CB1R) agonists, such as THC (Howlett et al. 2002; Varvel et al. 2005). The cannabinoid tetrad therefore serves as a useful tool to evaluate if a pharmacological compound is an agonist of the CB1R in rodents, indirectly testing for receptor function (Metna-Laurent et al. 2017a). Hence, our data indicate that the HIV-1 transgene does not influence standard THC-induced physiological effects, and likely does not impact CB1R function. These findings aid in the interpretation of past studies of THC-induced cognitive effects in the HIV-1tg rat model. Consistent with cannabis use effects observed in PWH (as reviewed by Ayoub et al. (2025)), we previously published function-dependent effects of THC on cognition in the HIV-1tg rat model (enhanced learning and riskier decision-making; (Ayoub et al. 2025)). Given the findings of the current study that THC-induced physiological and motivational effects were consistent across genotypes, the THC-induced cognitive effects described above can be more confidently be attributed to cognition rather that physiological responses, or CB1R function.

The therapeutic potential of cannabinoids for PWH may be produced through several mechanisms. First, chronic exposure to viral HIV-proteins drive neuroinflammatory processes in the central nervous system (CNS)(Young et al. 2022) which are thought to contribute to HIV-related NCI (Brody et al. 2025; Rackstraw 2011) and are tied to poorer functional outcomes in subpopulations of PWH (Thompson et al. 2022; Derry-Vick et al. 2022; Memiah et al. 2021; Rubin and Maki 2019). Cannabinoids, including THC, can reduce neuroinflammation (Kaddour et al. 2022; Yndart Arias et al. 2023) and may therefore alleviate inflammation-induced NCI. Second, the neuroprotective and neuroregenerative properties of cannabinoids (Aguado et al. 2007; Maccarrone et al. 2004) may counter HIV-induced neuronal toxicity and associated neural damage (Esposito et al. 2002; DeMarino et al. 2022). Hence, cannabinoids may reduce HIV-associated side effects through their well-known anti-inflammatory and neuroprotective properties.

While we tried to limit potential confounds, some limitations persist. First, the behavioral outcomes recorded were conducted after 0, 0.3 mg/kg and 3 mg/kg doses of THC only. Multiple studies utilize doses within this range (0.002 mg/kg – 3 mg/kg) and observed the attenuation of cognitive decline associated with aging (Bilkei-Gorzo et al. 2017; Suliman et al. 2018; Wang et al. 2022; Nidadavolu et al. 2021; Sarne et al. 2018). Further, the highest dose (3 mg/kg) represent the typical daily THC consumption in daily users (Borodovsky et al. 2025; Kerr and Ye 2022; Larsen et al. 2023). Past studies have conducted the cannabinoid tetrad in rodents at higher doses, including 10 mg/kg i.p. (Metna-Laurent et al., 2017a) and 1–20 mg/kg (oral gavage) (Moore and Weerts 2022). Our restricted dose range might affect the generalizability of results as dose-dependent effects and interactions with gene might occur at higher doses, although these doses match those used in cognitive studies. The final component of the cannabinoid tetrad, catalepsy, requires higher dosing, and thus not explored here. Nevertheless, we observed reproducible tetrad effect of antinociception, hypothermia, and hypolocomotion after 3 mg/kg THC consistent with a prior study in Sprague Dawley rats (Moore and Weerts 2022). It will be important for future work to utilize a broader range of THC doses to fully characterize dose-responsivity to tetrad effects across HIV-1tg rats and their controls. Future research should also investigate whether prolonged THC exposure leads to tolerance development, more akin to chronic cannabis use, and relevant to the possibility of cannabinoids as long-term therapeutics. Additional methods of administration should be considered for future studies to fully understand cannabinoid metabolism and pharmacokinetics in the HIV-1tg rat model. Cannabinoid content peaks and diminishes differently dependent on route of administration. For example, Hložek, Uttl (Hložek et al. 2017) discovered that while cannabinoid levels in serum and brain rapidly peaked and diminished after inhalation, subcutaneous and oral administration produced longer-lasting and higher peak levels. Furthermore, oral administration was reported to promote slower and more erratic absorption of THC (Grotenhermen 2003; Newmeyer et al. 2017; Wall et al. 1983; Moore et al. 2021). To our knowledge, no study to date has delivered cannabinoids to HIV-1tg rats via inhalation or oral routes, which should be prioritized by future studies given these are more consistent with consumption patterns found in humans and PWH. Finally, it would be worthwhile to test the co-administration of multiple cannabinoids such as ratios of THC and cannabidiol (CBD), the primary constituents of smoked cannabis that is consumed by PWH. CBD has been in use as a form of therapy for some seizures (Patra et al. 2019) and has been studied extensively for its neuroprotective properties (Barichello et al. 2012; El-Remessy et al. 2006; Iuvone et al. 2004; Mecha et al. 2012) and low abuse liability (Babalonis et al. 2017). Future research could benefit from exploring a broader range of THC doses, multiple modes of administration as well as explore co-administration of THC with other cannabinoids such as CBD to accurately compare behavioral outcomes with past and future studies.

## Conclusion

In summary, our study revealed that 3 mg/kg THC produced the tetrad (hypothermia, analgesia, and hypomotility) irrespective of HIV-1 genotype, and did not alter motivational deficits observed in the HIV-1tg rat model. Further research is needed to test a broader range of behaviors at both acute and chronic doses, explore different routes of administration, and investigate the co-administration of THC with other cannabinoids. By demonstrating that THC exerts comparable physiological effects across groups, these findings provide a basis for future studies assessing explicit cognitive domains, in which observed differences can be more confidently attributed to cognitive, rather than physiological, mechanisms. Overall, our work advances understanding of THC’s impact in the HIV-1tg model and supports continued investigation into the therapeutic potential of cannabis-based treatments for HIV-associated NCI.

## Supplementary Information

Supplementary Material 1.

## Data Availability

All data generated or analyzed during this study are included in this published article and its supplementary information files.

## Abbreviations

HIV: Human Immunodeficiency Virus
THC: Delta-9-tetrahydrocannabinol
HIV-1tg: HIV-1 transgenic
WT: Wildtype
F344: Fischer 344
BPM: Behavioral Pattern Monitor
PRBT: Progressive Ratio Breakpoint Task
PWH: People with HIV
NCI: Neurocognitive impairment
CNS: Central nervous system
CBD: Cannabidiol
eCB: Endocannabinoid
LWH: Length x width x height
UCSD: University of California, San Diego
IACUC: Institutional Animal Care and Use Committee
VEH: Vehicle
FR1: Fixed Ratio 1
TG: Transgenic
CB1: Type 1 cannabinoid receptor
iTat: Inducible Tat
SUD: Substance use disorder
