# Supplementary material for: THC induced similar physiological effects on HIV transgenic rats and their controls without affecting HIV-induced deficits in effortful motivation
Source: J Cannabis Res. 2026 Jan 2;8:17. doi: 10.1186/s42238-025-00383-8 (PMC12866464; doi:10.1186/s42238-025-00383-8)
Supplement: Supplementary file 1 — Supplementary Material 1. [file 42238_2025_383_MOESM1_ESM.docx]

Supplemental Figures

**Supplemental Figure 1: Sex interacted with group and THC in the tail flick assay and rectal temperature recordings, respectively.** Across all panels, left side graphs represent female data while right side graphs represent male data. Bar plots represent means, and individual points are shown ± S.E.M. Significant main effects of gene and sex are indicated by #p<0.1, **p<0.01, ***p<0.001. †= significant difference in group within dose. **ǂ** = significant difference in sex within group.

**A -** No main effect of sex was observed but sex interacted with group on tail flick time outcome in the tail flick assay F(2,115)=15.051, p<0.001] where F344 rats had lower tail flick times relative to the other groups in males only (ps<0.001). Further group comparisons revealed that males had higher tail flick time relative to females in WT (p<0.001) and TG (p=0.002) groups, whereas females had higher tail flick times relative to males within the F344 group (p<0.001).

**B -** No main effect of sex was observed on rectal temperature, but sex interacted with THC [F(2,115)=6.502, p=0.002]. Post hoc analyses revealed that 3 mg/kg THC reduced body temperature relative to 0.3 mg/kg THC and vehicle in females only (ps<0.001) while 3 mg/kg THC tended to reduce body temperature relative to 0.3 mg/kg THC in males (p=0.069). Further group comparisons revealed that males had lower body temperature relative to females when treated within VEH (p=0.012) or 0.3 mg/kg THC (trend; p=0.074), though females had lower temperatures than males when treated with 3 mg/kg THC. No main effect of sex or interactions of sex with group, or THC and group were observed (F<2.176, ns).

**S3 - Sex interacted with group and THC in some instances in the Behavioral Pattern Monitor (BPM).** Across all panels, left side graphs represent female data, right side graphs represent male data, and significance bars between these graphs refer to main effects if sex. Bar plots represent means, and individual points are shown ± S.E.M. Significant main effects are indicated by, *p<0.05, ***p<0.001. †= significant difference in group within dose **£** = significant effect of sex within dose

**A -** A main effect of sex was observed on counts in the BPM [F(1,115)=17.827, p<0.001], with post hoc analyses revealing that females had higher counts compared to males.

**B -** A main effect of sex was observed on transitions in the BPM [F(1,115)=15.338, p<0.001], with post hoc analyses revealing that females had higher transitions than males. Additionally, a group and sex interaction was observed [F(2,115)=2.575, p=0.081], with post hoc analyses revealing that WT rats had higher transitions relative to TG rats in females only (p=0.045). Further group comparisons revealed that females had higher transitions than males in WT (p<0.001) and TG (p=0.015) groups only.

**C -** A main effect of sex was observed on distance travelled in the BPM [F(1,115)=14.606, p<0.001], with post hoc analyses revealing that females travelled a higher distance relative to males. A trending group x sex interaction was also observed [F(2,115)=2.438, p=0.092], with post hoc analyses revealing that WT rats travelled higher distances relative to TG rats in females only (p=0.040). Further group comparisons revealed that females travelled higher distances than males in WT (p<0.001) and TG (p=0.017) groups only.

**D -** A main effect of sex was observed on pokes in the BPM [F(1,115)=54.159, p<0.001], with post hoc analyses revealing that females had higher pokes compared to males. The effect of THC appeared dependent on sex [F(2,115)=3.396, p=0.037], with post hoc analyses revealing that 3 mg/kg THC reduced pokes relative to 0.3 mg/kg THC (p=0.002) and vehicle (p=0.008) in females only. Further group comparisons revealed that males had decreased pokes relative to females across VEH and THC doses (ps<0.05-0.001). No main or interactive effects of group were observed (F<2.254, ns).

**E -** No main or interactive effects of sex were observed on rearing behavior in the BPM (F<1.695, ns).

**F -** A main effect of sex was observed on spatial d in the BPM [F(1,115)=6.520, p=0.012], with post hoc analyses revealing that females had higher spatial d relative to males. No interaction between group and sex was seen (F<2.176, ns).
